# Supplementary material for: A Possible Way to Relate the Effects of SARS-CoV-2-Induced Changes in Transferrin to Severe COVID-19-Associated Diseases
Source: Int J Mol Sci. 2022 May 31;23(11):6189. doi: 10.3390/ijms23116189 (PMC9181396; doi:10.3390/ijms23116189)
Supplement: Supplementary file 1 [file ijms-23-06189-s001.zip › ijms-1715157-supplementary.pdf]

Supplementary materials

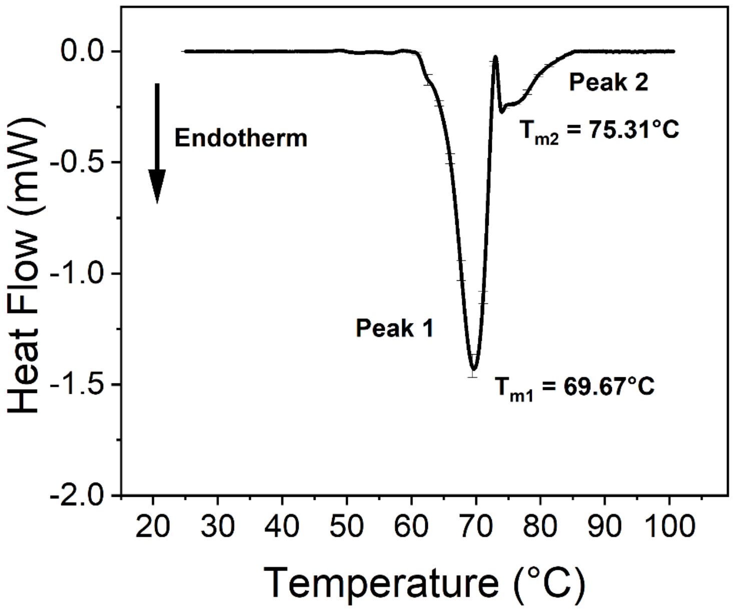

Figure S1. Thermal analysis of virus-treated anticoagulated whole blood samples after 2 hours of incubation. The plot is an average of the same measurements repeated at different incubation temperatures (24 °C, 37 °C and 40 °C). The DSC data represent the mean ± SD of three independent measurements (n = 3).

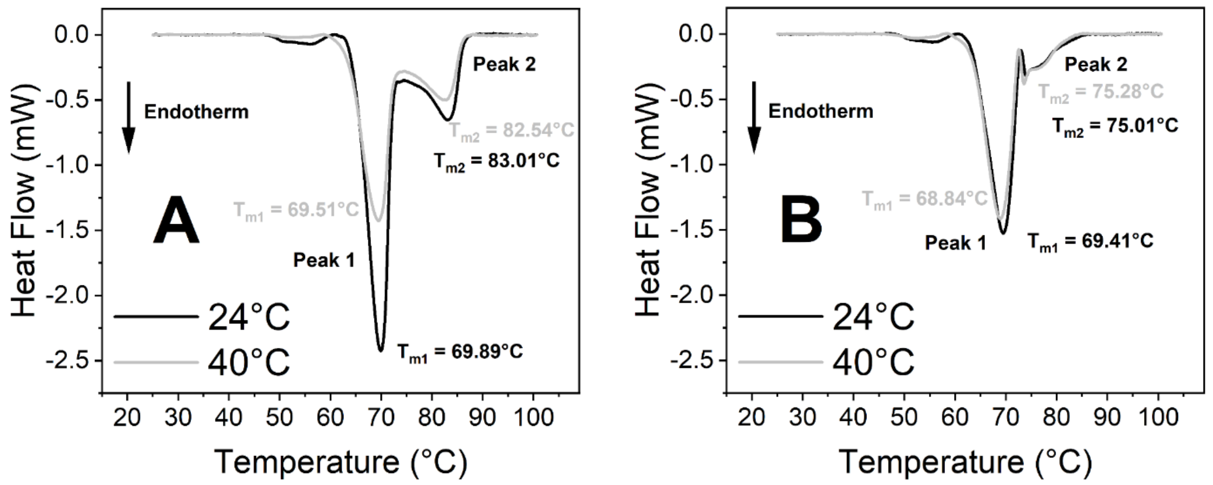

Figure S2. Thermal analysis of control (**A**) and virus-treated (**B**) anticoagulated whole blood samples after 50 hours of incubation at 24 °C and at 40 °C.

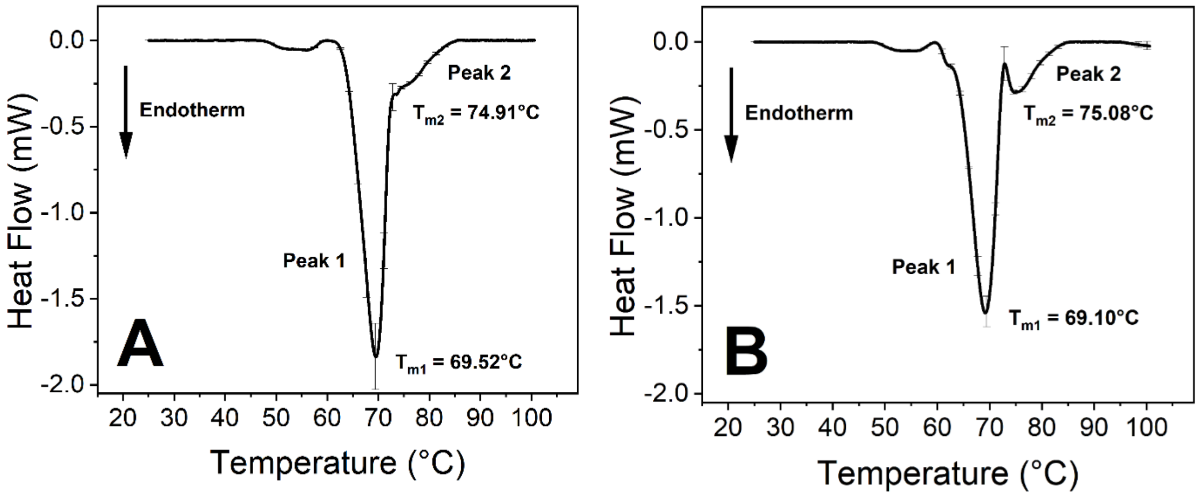

Figure S3. Thermal analysis of control (A) and virus-treated (B) anticoagulated whole blood samples after 15 hours of incubation at 37 °C. The DSC data represent the mean ± SD of four independent measurements (n = 4).

Table S1. Iron metabolism-related parameters of healthy whole blood. Data represent mean ± SD.

**Iron Metabolism-Related Blood Parameters of Healthy Volunteers**		
**Iron level** (μmol/L)	**Transferrin** (g/L)	**Ferritin** (μg/L)
21.65 ±2.20	2.80 ± 0.67	143.50 ± 21.92
